# The active functional microbes contribute differently to soil nitrification and denitrification potential under long-term fertilizer regimes in North-East China

**DOI:** 10.3389/fmicb.2022.1021080

**Published:** 2022-10-03

**Authors:** Feng Wang, Xiaolong Liang, Fan Ding, Lingling Ren, Minjie Liang, Tingting An, Shuangyi Li, Jingkuan Wang, Lingzhi Liu

**Affiliations:** ^1^Key Laboratory of Arable Conservation in Northeast China, Ministry of Agriculture and Rural Affairs, College of Land and Environment, Shenyang Agricultural University, Shenyang, China; ^2^Key Laboratory of Pollution Ecology and Environmental Engineering, Institute of Applied Ecology, Chinese Academy of Sciences, Shenyang, China

**Keywords:** functional gene, transcription, fertilization regimes, nitrification, denitrification

## Abstract

Nitrogen (N) cycling microorganisms mediate soil nitrogen transformation processes, thereby affecting agricultural production and environment quality. However, it is not fully understood how active N-cycling microbial community in soil respond to long-term fertilization, as well as which microorganisms regulate soil nitrogen cycling in agricultural ecosystem. Here, we collected the soils from different depths and seasons at a 29-year fertilization experimental field (organic/chemical fertilizer), and investigated the transcriptions of N-cycling functional genes and their contribution to potential nitrification and denitrification. We found that long-term fertilization exerted significant impacts on the transcript abundances of nitrifiers (AOA *amo*A, AOB *amo*A and *hao*) and denitrifiers (*nar*G and *nos*Z), which was also notably influenced by season variation. The transcriptions of AOA *amo*A, *hao*, and *nar*G genes were lowest in autumn, and AOB *amo*A and *nos*Z transcript abundances were highest in autumn. Compared to no fertilization, soil potential nitrification rate (PNR) was reduced in fertilization treatments, while soil potential denitrification rate (PDR) was significantly enhanced in organic combined chemical fertilizer treatment. Both PNR and PDR were highest in 0–20 cm among the tested soil depths. Path model indicated active nitrifiers and denitrifiers had significant impact on soil PNR and PDR, respectively. The transcriptions of AOA *amo*A and *nxr* genes were significantly correlated with soil PNR (Pearson correlation, *r > 0.174*, *p < 0.05*). Significant correlation of *nap*A and *nos*Z transcriptions with soil PDR (Pearson correlation, *r > 0.234*, *p < 0.05*) was also revealed. Random forest analysis showed that SOC content and soil pH were the important factors explaining the total variance of active nitrifers and denitrifiers, respectively. Taken together, long-term fertilization regimes reduced soil PNR and enhanced PDR, which could be attributed to the different responses of active N-cycling microorganisms to soil environment variations. This work provides new insight into the nitrogen cycle, particularly microbial indicators in nitrification and denitrification of long-term fertilized agricultural ecosystems.

## Introduction

Nitrogen (N) fertilizer application can facilitate rapid and effective N uptake by crops, playing an irreplaceable role in maintaining soil fertility and promoting crop growth. The application of N fertilizer has increased in recent decades to meet the needs of crop production around the world (Erisman et al., 2008; Hayden et al., 2010). However, the overuse of N fertilizers might break the balance of N transformation in soils, because of the accumulation and leaching of soil nitrate, excessive emission of N_2_O, and so on, and cause a series of serious ecological problems, such as biodiversity loss, soil degeneration, and environmental pollution (Vitousek et al., 1997; Wang et al., 2016). As one of the central element cycles in soil ecosystems, N cycle is driven by soil microorganisms, transferring nitrogen into, within, and out of soils *via* biological processes of nitrification, denitrification, dissimilatory nitrate reduction to ammonium (DNRA), N_2_ fixation, and ammonification (Vitousek et al., 1997; Luo et al., 2018). The functional gene markers of soil N-cycling microorganisms are generally used to describe their abundance, activity, and diversity (Jetten, 2008; Nelson et al., 2015).

It is commonly believed that fertilization could directly influence N-cycling microorganisms and the expression of their functional genes, thus impacting nitrogen biogeochemical processes profoundly (Shen et al., 2008; Bandyopadhyay et al., 2010). Previous reports showed that chemical fertilizers can negatively impact the abundances of ammonia oxidizers (AOA and AOB) and denitrifiers, inhibiting soil potential nitrification and denitrification (Wakelin et al., 2007; Fan et al., 2011). Compared to chemical fertilizers, organic fertilizers may increase the abundances of nitrifiers and *nos*Z-containing denitrifiers at both DNA and RNA level, which promotes the activity of soil nitrification and denitrification (Chen et al., 2012; Wang et al., 2014; Zhe et al., 2015). However, a study by Wang et al. (2018) showed that chemical fertilizers positively affected soil denitrification rates in a terrestrial ecosystem. Indeed, other environmental factors such as seasonal variations and soil depth may also influence soil N-cycling microorganisms (Shaver et al., 2000; Stewart et al., 2012; Liang et al., 2020), even playing bigger roles than fertilization (Kathryn et al., 2012; Wang et al., 2016). For example, Cindy et al. (2015) examined the denitrification and DNRA communities in sediment samples and revealed that the abundance of *nap*A- and *nar*G-containing denitrifiers and *nrf*A-containing DNRA microorganisms were higher in June or August than in other months, with the rates of both denitrification and DNRA being highest in August. Waghmode et al. (2018) examined the response of soil microbial community structure to warming and soil depth at North China Plain, and their results suggested that soil potential nitrification rates and AOB abundance were promoted in warming treatments. However, warming decreased the abundances of AOA and denitrifiers (*nir*K, *nir*S, and *nos*Z), bacterial diversity and species richness, with the effect being more significant in surface soil than in deep soil. However, the relative and interactive contribution of fertilization, seasonality, and soil depth to specific N transformation process in agroecological system of north-east China is still poorly understood.

Soil properties can profoundly impact N-cycling, including nitrificaition and denitrification (Alldred and Baines, 2016). For example, Dong et al. (2009) revealed the soil potential nitrification rates in a corn field were increased by an increase in NH_4_^+^ availability. Soil denitrification and DNRA rates were shown to be linked to nitrate concentrations, because the latter can be used as substrates for the former (Peng et al., 2016). Sun et al. (2012) indicated that the soil denitrification rates and N_2_/N_2_O emissions were mainly controlled by soil pH in forest and grassland soils. It remains unclear whether environmental factors affect soil nitrogen cycling directly or indirectly through functional microorganisms. Comprehensive analysis of soil properties and key functional microorganisms associated with various processes of the soil nitrogen cycling based on functional gene transcripts is vital for better understanding the ecological contribution of N-cycling microorganisms in soil ecosystems.

This study aimed to examine the impacts of long-term fertilization, seasonal variation, and soil depth on N cycling activities and estimate the influence by related active microbial communities and different environmental factors. Soil samples were collected from an agricultural site with different long-term fertilization regimes, i.e., N chemical fertilizer and manure fertilizer combined with N chemical fertilizer. We investigated the N-cycling rates (potential nitrification and denitrification) and used specific genes as markers to quantify different active N-cycling microbial groups, including *amo*A (encoding ammonia monooxygenase), *nxr* (encoding nitrite oxidoreductase), and *hao* (encoding hydroxylamine oxidoreductase) for nitrification (Poly et al., 2008; Rasche et al., 2011; Kuypers et al., 2018); and *nap*A and *nar*G (encoding nitrate reductase), *nir*K and *nir*S (encoding nitrite reductase), *nor*B (encoding nitric oxide reductase), and *nos*Z (encoding nitrous oxide reductase) for denitrification (Smith et al., 2007; Luo et al., 2018). We hypothesized that: (1) interactive effect of fertilization, seasonality, and soil depth on N-cycling microorganisms and N transformation rates would be greater than individual effect of these factors; and (2) environmental factors would affect soil nitrogen transformation processes through regulating relevant microorganisms.

## Materials and methods

### Site description and soil sampling

The long-term experimental site located in Shenyang Agricultural University (41°49′ N, 123°34′ E), Liaoning Province, China. The mean annual temperature is 7.9°C, and the mean annual rainfall is 705 mm. The soil texture is Hapli-Udic Alfisol according to the USDA Taxonomy (Soil Survey Staff, 1999). The site has been used for maize (*Zea mays* L.) monoculture for more than 29 years, including the following four fertilization treatments: (1) CK, unfertilized control, (2) LCF, urea chemical fertilizer consisting of 135 kg N ha ^−1^, (3) HCF, urea chemical fertilizer consisting of 270 kg N ha ^−1^, and (4) CMF, LCF plus 135 kg N ha ^−1^ pig compost. The pig compost consisted of 150 g kg ^−1^ organic carbon, 10 g kg ^−1^ N, 4.4 g kg ^−1^ phosphorus, and 3.3 g kg ^−1^ potassium on a dry weight basis.

In each fertilization site, soil samples with different seasons (spring, summer, and autumn) were collected from a vertical profile with 60 cm that corresponded to depths of 0–20, 20–40, and 40–60 cm. Each soil composite sample was a mixture of the five soil cores for a given soil layer. After removing visible stones, crop roots, and debris, soil sample was shipped on ice to the laboratory and divided into two subsamples. One subsample was stored at −80°C for the RNA extraction, and the other was stored at 4°C for the soil properties analysis.

### Soil chemical properties determination

The soil chemical properties were determined by the following methods (Ling et al., 2014). Soil pH was analyzed with a soil to water ratio of 1: 2.5 using a pH meter (Metrohm 702, Switzerland). Soil organic carbon (SOC) and total nitrogen (TN) were determined *via* the Dumas combustion method on an elemental analyzer (Elementar Vario MAX cube, Germany). Soil ammonia (NH_4_^+^-N) and nitrate (NO_3_^−^-N) were extracted by 2 M KCl solution, filtered and analyzed with a continuous flow analyzer (AA3-HR, SEAL, Germany) as described in Bai et al. (2012).

### Soil RNA extraction and quantitative PCR analysis

Total RNA was extracted from 0.5 g of soil sample using E.Z.N.A.® Soil RNA Kit (OMEGA, United States) according to the manufacturer’ s instructions. The detection of RNA purity was confirmed with 1% agarose gel electrophoresis, and the concentrations of RNA extractions were determined with a NanoDrop ND-2000 spectrophotometer (NanoDrop Technologies; Thermo Scientific, United States). The first strand of cDNA was synthesized with high efficiency reverse transcriptase using HiFiScript cDNA Synthesis Kit (CWBIO, China). The RNA and cDNA samples were stored at −80°C for subsequent analyses.

All quantitative PCR (qPCR) assays were performed on an ABI Step One Plus Real-time PCR System (Applied Biosystems, United States). Detected genes included archaeal and bacterial *amo*A, *hao*, and *nxr* genes in nitrification and *nar*G, *nap*A, *nor*B, *nos*Z, *nir*S, and *nir*K genes in denitrification. The primer sets for quantification of these genes are shown in Supplementary Table S1. The 20-μl reaction mixture contained 10 μl of SYBR ® Premix Ex Taq (2x; Tli RnaseHPlus), 0.5 μl of each primer (10 μM), 2 μl of template cDNA, and 7 μl ddH_2_O to bring the final volume up to 20 μl. Negative controls were performed in which template cDNA was replaced by ddH_2_O. The amplified condition were an initial denaturation step at 95°C for 10 min, followed by 40 cycles of denaturation at 95°C for 15 s, annealing at 55–60°C for 30 s, and extension at 72°C for 30–60 s. Standard curves were generated using 10-fold serial dilutions of plasmids containing correct inserts of target genes. Melting curve was used to check the products specificity at the end of each qPCR run. The amplification efficiencies of all detected genes were 90.25–100.86%, and the *R*^2^ values were > 0.98. Each sample was conducted in three technical replicates, and the gene transcripts of three replicates were normalized by the copy numbers in per gram of dry soil used in each sample.

### Potential soil N-cycling rates determination

Soil PNR was determined according to the procedures described by He et al. (2007) with minor modifications: 5.0 g of fresh soil sample was added to 100 ml flasks containing 1 M phosphate buffer solution with 1.5 mM (NH_4_)_2_SO_4_. After incubation at 25°C for 14 days in darkness, 25 ml of 1 M KCl solution were added to the flask and the nitrate (NO_3_^−^-N) concentrations in the supernatant were measured immediately. The PNR is expressed as the amount of NO_3_^−^-N produced per unit of time.

Soil PDR was determined according to Delaune et al. (1998) and Reddy et al. (1980) with minor modifications: 5.0 g of fresh soil sample was added to 100 ml flasks containing 7.5 ml deionized water preincubating 2 days, which could create a flooded anaerobic condition, conduciving to the soil denitrification (Xu and Cai, 2007). Flasks were added 3.6 g L^−1^ KNO_3_ incubating at 25°C for 14 days in darkness. 25 ml of 1 M KCl solution was added to the flask and the nitrate (NO_3_^−^-N) concentrations in the supernatant were measured immediately. The PDR is expressed as the amount of NO_3_^−^-N loss per unit of time.

### Statistical analyses

Significant differences and attributing variable inside a data set were analyzed with one-way ANOVA, and the least significant difference (LSD) was used to compare the means for each variable using SPSS (IBM Software, United States, version 20.0). All statistical tests were considered significance at *p < 0.05*.

To evaluate the responses of the soil active N-cycling microorganisms to fertilizers, soil horizons, and seasons, principal component analysis (PCA) and permutational multivariate ANOVA (PERMANOVA) were implemented using the Vegan package of R (version 3.4.4). Pearson correlation coefficients using SPSS (version 20.0) and classification random forest (RF) analysis using the randomForest packages of R (version 3.4.4) were performed to investigate the relationship between active N-cycling microorganisms and soil properties. In the random forest analysis, the higher percentage of the mean squared error (MSE%) values of variables imply more important variables, and the significance of the models and variables were assessed with the A3 package and rfPermute package, respectively.

To evaluate the direct and indirect effects of soil properties on soil N-cycling rates, partial least square path model (PLS-PM) was conducted using PLSPM packages of R (version 3.4.4). Goodness of fit (GoF) index was used to assess the overall fit degree of the model, and the larger the GoF value, the higher the model fit degree (Liao et al., 2018). The conceptual path model was developed according to Ma et al. (2020).

## Results

### Soil chemical properties across fertilization, soil horizon, and season

Soil chemical properties were significantly influenced by fertilization (Table 1). In general, fertilization significantly increased soil NH_4_^+^ and NO_3_^−^ concentrations (*p < 0.05*) and decreased soil pH and C/N ratios. The soil properties also showed significant differences among different fertilization treatments (LCF, HCF, and CMF). Specifically, NH_4_^+^ and NO_3_^−^ concentrations in HCF treatment were significantly higher than those in LCF and CMF treatments (*p < 0.05*), while soil pH value and the contents of SOC and TN were notably higher in CMF treatment than N chemical fertilizer (LCF and HCF) treatments (*p < 0.05*).

**Table 1:** Effects of fertilization, soil horizon, and season on soil chemical properties.

	SOC (g kg^−1^)	TN (g kg^−1^)	C/N	pH	NH_4_^+^ (mg kg^−1^)	NO_3_^−^ (mg kg^−1^)
Fertilization (F)						
CK	7.39 b	0.75 b	9.82 a	5.97 a	0.64 c	6.19 c
LCF	7.25 b	0.77 b	9.39 ab	5.31 b	1.23 b	21.29 ab
HCF	7.04 b	0.77 b	9.11 b	5.16 b	1.65 a	22.12 a
CMF	8.40 a	0.89 a	9.28 ab	5.86 a	1.30 b	15.71 b
Soil horizon (H)						
0–20 cm	10.71 a	1.05 a	10.25 a	5.30 b	1.93 a	16.95 a
20–40 cm	6.68 b	0.67 b	9.98 a	5.72 a	1.04 b	16.49 b
40–60 cm	5.17 c	0.66 b	7.97 b	5.71 a	0.90 b	15.55 c
Season (S)						
Spring	7.41	0.79	9.26	5.46 b	1.86 a	12.48 b
Summer	7.53	0.78	9.34	5.43 b	1.14 b	26.62 a
Autumn	7.62	0.81	9.60	5.84 a	0.87 b	9.89 c
ANOVA(*p* value)						
Fertilization	**<0.001**	**<0.001**	**0.002**	**<0.001**	**<0.001**	**<0.001**
Soil horizon	**<0.001**	**<0.001**	**<0.001**	**<0.001**	**<0.001**	**<0.001**
Season	0.086	0.119	0.092	**<0.001**	**<0.001**	**<0.001**
F*H	**<0.001**	**<0.001**	**0.008**	**<0.001**	**<0.001**	**<0.001**
F*S	0.053	0.587	0.929	**<0.001**	**<0.001**	**<0.001**
H*S	0.069	0.659	0.508	**0.008**	**<0.001**	**<0.001**
F*H*S	**0.010**	0.242	0.975	**<0.001**	**<0.001**	**<0.001**

a SOC, soil total carbon; TN, soil total nitrogen. C/N, The ratio of SOC to TN.

b Different letters indicate significant difference for main effect of the three treatments based on LSD multiple comparisons test (*p* < 0.05).The bold values represent significance at 0.05 level.

Soil chemical properties also varied exceedingly in different soil horizons and seasons (Table 1). The values of tested soil chemical properties, except soil pH, were reduced with decreasing depth at horizons. Soil pH was significantly higher in the 20–40 and 40–60 cm soil layers than that in the 0–20 cm soil layer (*p < 0.05*). Soil samples in spring had higher NH_4_^+^ concentrations (*p < 0.05*) than those in summer and autumn. The NO_3_^−^-N concentration was the highest in summer, while soil pH was highest in autumn (*p < 0.05*). Interestingly, the soil TN and SOC contents had no significant difference between different seasons.

### Active N-cycling microorganisms across fertilization, soil horizon, and season

**Figure 1:**
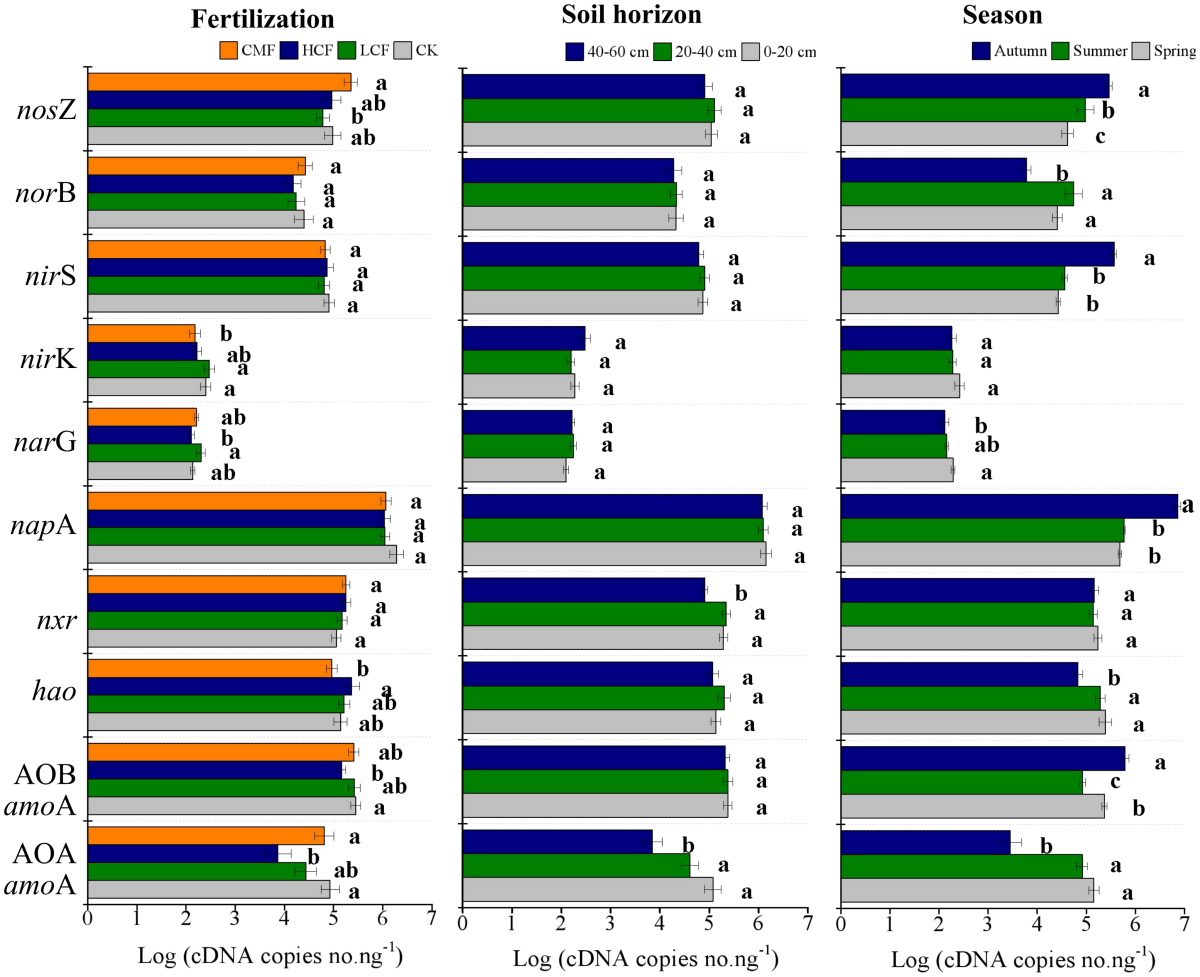
Variation in active genes in nitrification and denitrification as influenced by fertilization, soil horizon, and season. The letters represent significant differences at *p* ≤ 0.05 according to the LSD multiple comparisons test in different treatments. CK, control without fertilizers; LCF, low N chemical fertilizer; HCF, high N chemical fertilizer; and CMF, manure fertilizer combined with N chemical fertilizer.

Fertilizer significantly influenced the abundances of AOA *amo*A, AOB *amo*A, *nir*K and *nos*Z transcripts compared with no fertilization (*p < 0.05*; Figure 1). In nitrification, the transcript abundances of AOA and AOB *amo*A genes in high chemical fertilizers were significantly reduced by 21.70 and 5.32% respectively, (*p < 0.05*), compared to those in the CK samples. The active *hao* gene showed the opposite results in the same treatments compared to AOA and AOB *amo*A genes. In denitrification, the abundance of both *nar*G and *nos*Z transcripts was significantly increased by 2.34 and 7.43% in CMF treatment, respectively (*p < 0.05*), compared to CK treatment.

Although soil depths showed limited effect on the N-cycling genes (Figure 1), the effect of fertilizer on active N-cycling microbial community was more pronounced in surface soil. Among the tested N-cycling genes, only the active nitrifiers groups of AOA *amo*A and *nxr* were significantly different across the examined soil horizons (*p < 0.05*; Figure 1). The abundance of AOA *amo*A transcripts in the 0–20 and 20–40 cm soil layers were 32.03 and 20.05% higher than that in the 40–60 cm soil layer, respectively. In 0–20 cm soil layer, chemical fertilizer (LCF and HCF) treatments reduced the abundance of AOA *amo*A, *nar*G, and *nos*Z transcripts, CMF treatment reduced the abundance of *nir*K transcripts, but increased *nxr* transcripts significantly (Supplementary Figure S1; *p < 0.05*), compared to CK treatment.

Seasonal variation affected the N-cycling genes significantly, and the impacts of fertilization on active N-cycling microbial community were clearly different across seasons (Supplementary Figure S2). Almost all active nitrifiers and denitrifrers (except *nxr* in nitrification and *nirK* in denitrification) varied significantly in different seasons (*p < 0.05*; Figure 1). The transcriptions of AOA *amo*A, *hao, nar*G, and *nor*B was lowest in autumn, but the abundances of AOB *amo*A, *nap*A, *nir*S, and *nos*Z transcripts were highest in autumn, among the three seasons (*p < 0.05*). Compared to CK treatment, chemical fertilizer (LCF and/or HCF treatments) significantly increased the transcript abundance of AOB *amo*A and *nir*K genes in spring, *hao* gene in summer and *nar*G and *nir*S genes in autumn (*p < 0.05*), and CMF treatment increased the transcript abundance of *nos*Z gene in both spring and summer (*p < 0.05*).

### Potential nitrification and denitrification rates across fertilization, soil horizon, and season

**Figure 2:**
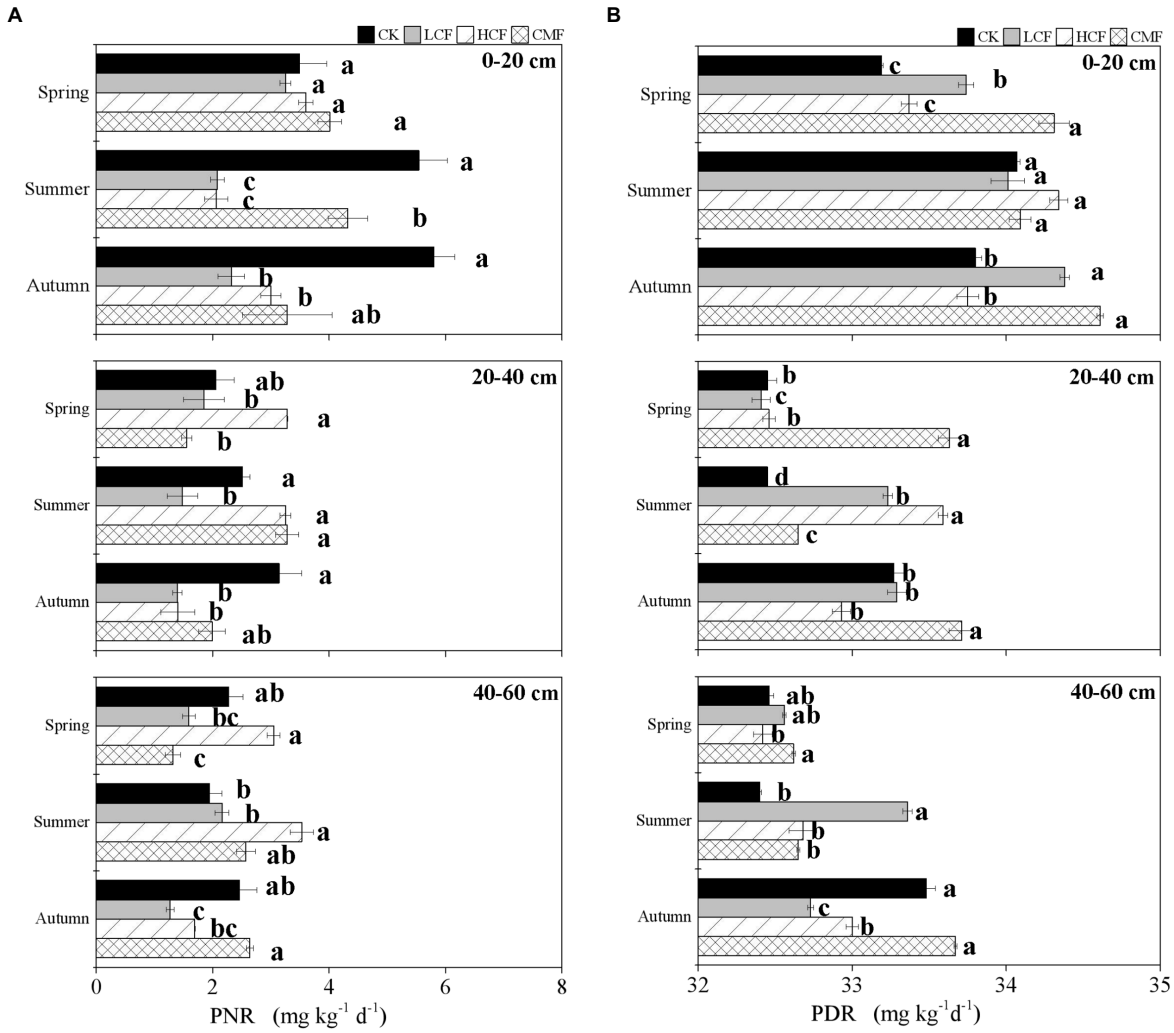
Variation in N-cycling rates as influenced by fertilization, soil horizon and season. **(A)** The changes of soil potential nitrification rate (PNR). **(B)** The changes of soil denitrification rate (PDR). The letters represent significant differences between samples (*p <* 0.05).

The soil PNR ranged from 0.41 to 6.99 mg kg^−1^ day^−1^ (Figure 2A). Compared to the CK treatment, soil PNR was significantly reduced by 40.43% in LCF treatment (*p < 0.05*), and lower PNR was also observed in HCF and CMF treatments (the differences were not statistically significant; Supplementary Table S2). Soil PNR was significantly higher in the surface horizon (0–20 cm) than those in the other soil horizons (*p < 0.05*). Compared to the soil PNR in 0–20 cm soil layer, the soil PNR in the 20–40 and 40–60 cm soil layers was 36.52 and 37.92% lower, respectively. Soil PNR was not significantly different across different seasons. Specifically, chemical fertilizer (LCF and/or HCF treatments) significantly reduced soil PNR at 0–20 and 20–40 cm soil layers in summer and autumn and at 40–60 cm soil layer in autumn, but CMF treatments only significantly reduced PNR at 0–20 cm soil layer in summer and at 40–60 cm soil layer in spring (*p < 0.05*; Figure 2A).

The soil PDR ranged from 32.19 to 34.68 mg kg^−1^ day^−1^ (Figure 2B). Compared to the CK treatment, soil PDR was significantly increased in CMF treatment (*p < 0.05*; Supplementary Table S2). Similar to soil PNR, soil PDR was higher in 0–20 cm soil layer than in the other soil horizons (*p < 0.05*). Soil PDR was lower in spring than in summer and autumn (*p < 0.05*), which was different from the trend of soil PNR. Specifically, CMF treatments significantly increased soil PDR at 0–20 and 20–40 cm soil layers in spring and autumn (*p < 0.05*). Chemical fertilizer (LCF and/or HCF treatments) increased soil PDR at 0–20 cm soil layer in spring and autumn and at 20–40 and 40–60 cm soil layers in summer, but PDR was reduced by chemical fertilizer at 20–40 cm soil layer in spring and at 40–60 cm soil layer in autumn (*p <* 0.05; Figure 2B).

### Relative importance of environmental factors In influencing active N-cycling microorganisms

**Figure 3:**
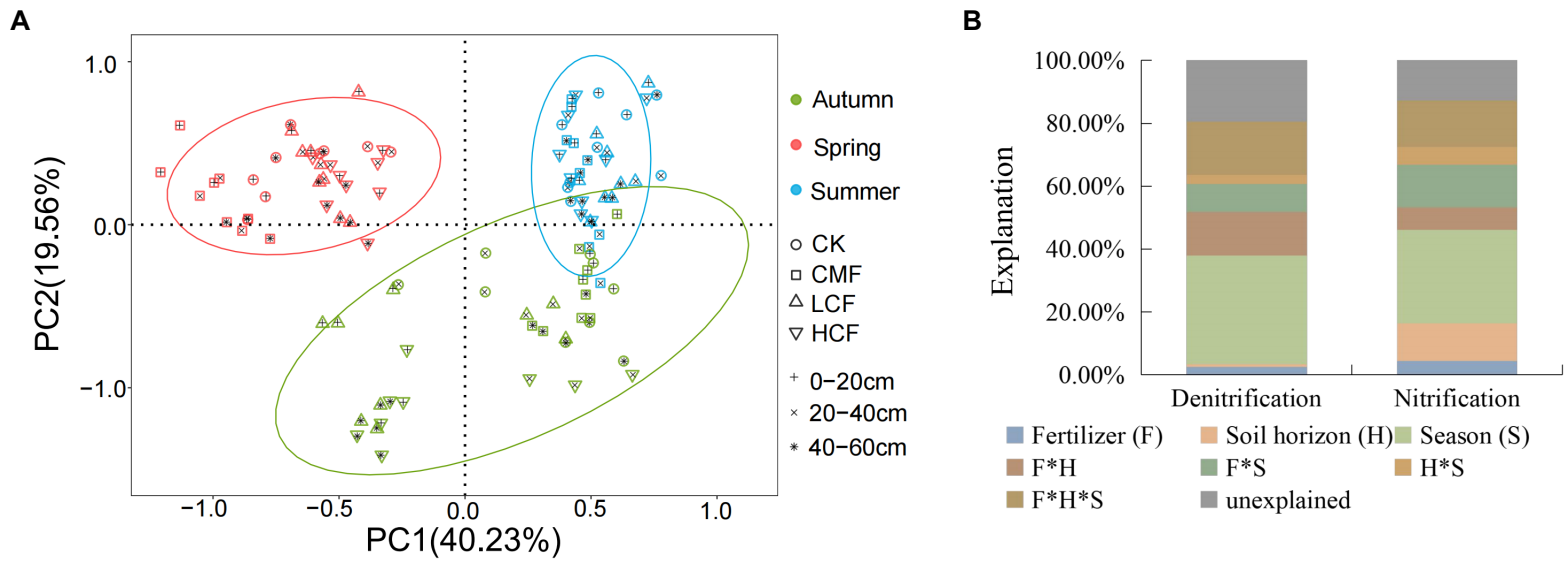
Comparison of individual and interactive effects of fertilization, soil horizon, and season on active N-cycling microorganisms. **(A)** Principal component analysis (PCA) of active N-cycling microorganisms. **(B)** Permutational multivariate ANOVA (PERMANOVA) of nitrifiers and denitrifiers. CK, control without fertilizers; LCF, low N chemical fertilizer; HCF, high N chemical fertilizer; and CMF, manure fertilizer combined with N chemical fertilizer.

The principal component analysis (PCA) revealed that the microbial abundances in nitrification and denitrification from different seasons were clearly separated. Soil samples from spring were clustered and located on the right side of the second axis (PC2) showing notable differences compared with the summer and autumn samples (Figure 3A). PERMANOVA (Figure 3B) indicated that season had the strongest effects on the transcription of microbial genes involved in nitrification and denitrification, accounting for 29.73 and 34.46% of the variations in functional gene transcript abundances (Supplementary Table S3; *p <* 0.01) respectively. Fertilizer contributed 4.50 and 2.54% to the changes in the transcription of nitrifiers and denitrifiers genes, respectively, and the interaction of fertilizer, season and soil horizon explained 14.76 and 16.84% of variations in the corresponding functional groups, respectively. Besides that, all the nitrifiers and denitrifiers (except for *nor*B) were significantly affected by fertilizer (*p < 0.05*), while the abundances of *nir*K, *nxr* and AOB *amo*A transcripts were not affected by the interaction of fertilizer, soil horizon and/or season (*p* > 0.05; Supplementary Table S3).

**Figure 4:**
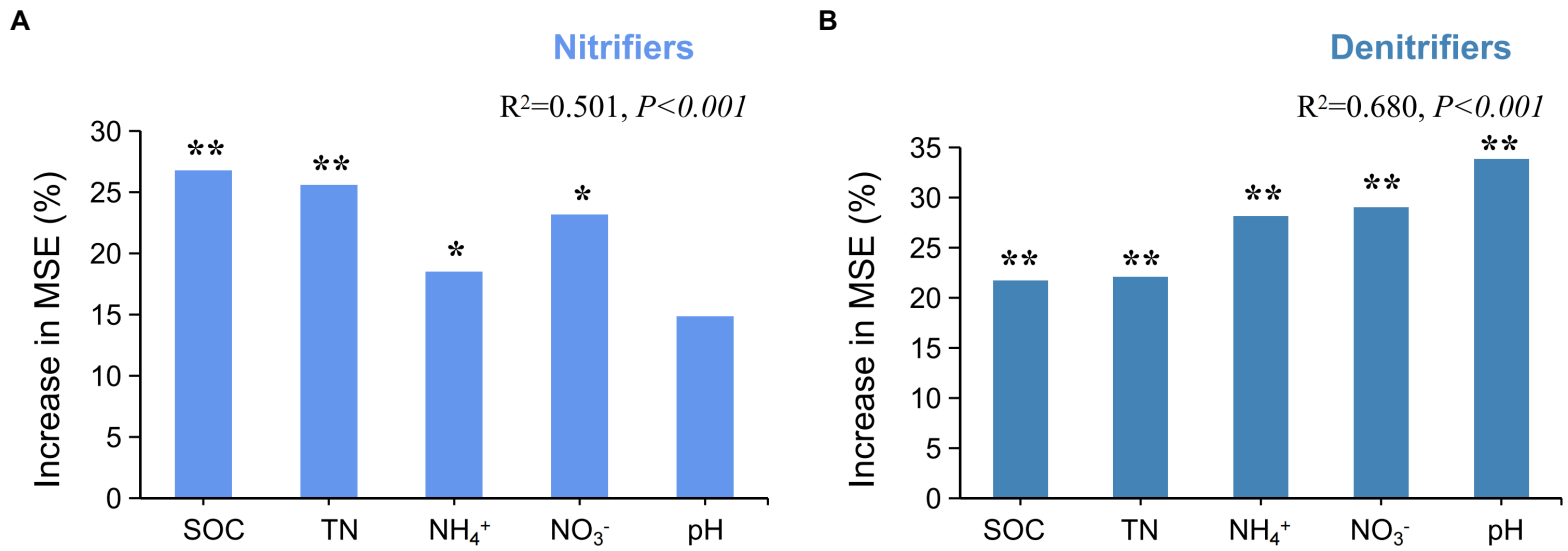
Effects of soil chemical factors on active N-cycling microorganisms. **(A)** Random forest analysis of nitrifiers and soil chemical factors. **(B)** Random forest analysis of denitrifiers and soil chemical factors. * and ** represent significance of variables at 0.05 and 0.01 level, respectively.

Random forest (RF) analysis was performed to investigate the potential main drivers of active N-cycling microorganisms. The results showed that SOC and TN were the main factors influencing active nitrifiers (*p <* 0.01; Figure 4A). Pearson’s correlation analysis also showed that SOC and TN had significant positive correlations with active AOA *amo*A and *nxr* (*p <* 0.01; Supplementary Table S4). The RF results also showed that soil pH, NO_3_^−^, and NH_4_^+^ were the main factors influencing active denitrifiers (*p <* 0.01; Figure 4B). Soil pH had significant positive correlation with *nap*A, *nir*S, and *nos*Z (*p <* 0.01). Soil NH_4_^+^ and NO_3_^−^ had significant negative correlations with both *nap*A and *nir*S (*p <* 0.01; Supplementary Table S4).

### Relative importance of environmental and biological factors in influencing potential N-cycling rates

Both of soil PNR and PDR were positively associated with soil SOC (*r* = 0.554 and *r* = 0.754 respectively; *p <* 0.01; Supplementary Table S4) and TN (*r* = 0.550 and *r* = 0.720 respectively; *p <* 0.01), while soil NH_4_^+^ and NO_3_^−^ were only positively correlated with soil PDR (*r* = 0.303 and *r* = 0.229, respectively; *p <* 0.05). Moreover, soil PNR was positively correlated with the abundance of AOA *amo*A and *nxr* transcripts, and soil PDR was positively correlated with the abundance of *nap*A, *nir*S and *nos*Z transcripts (*p <* 0.05; Supplementary Table S4).

**Figure 5:**
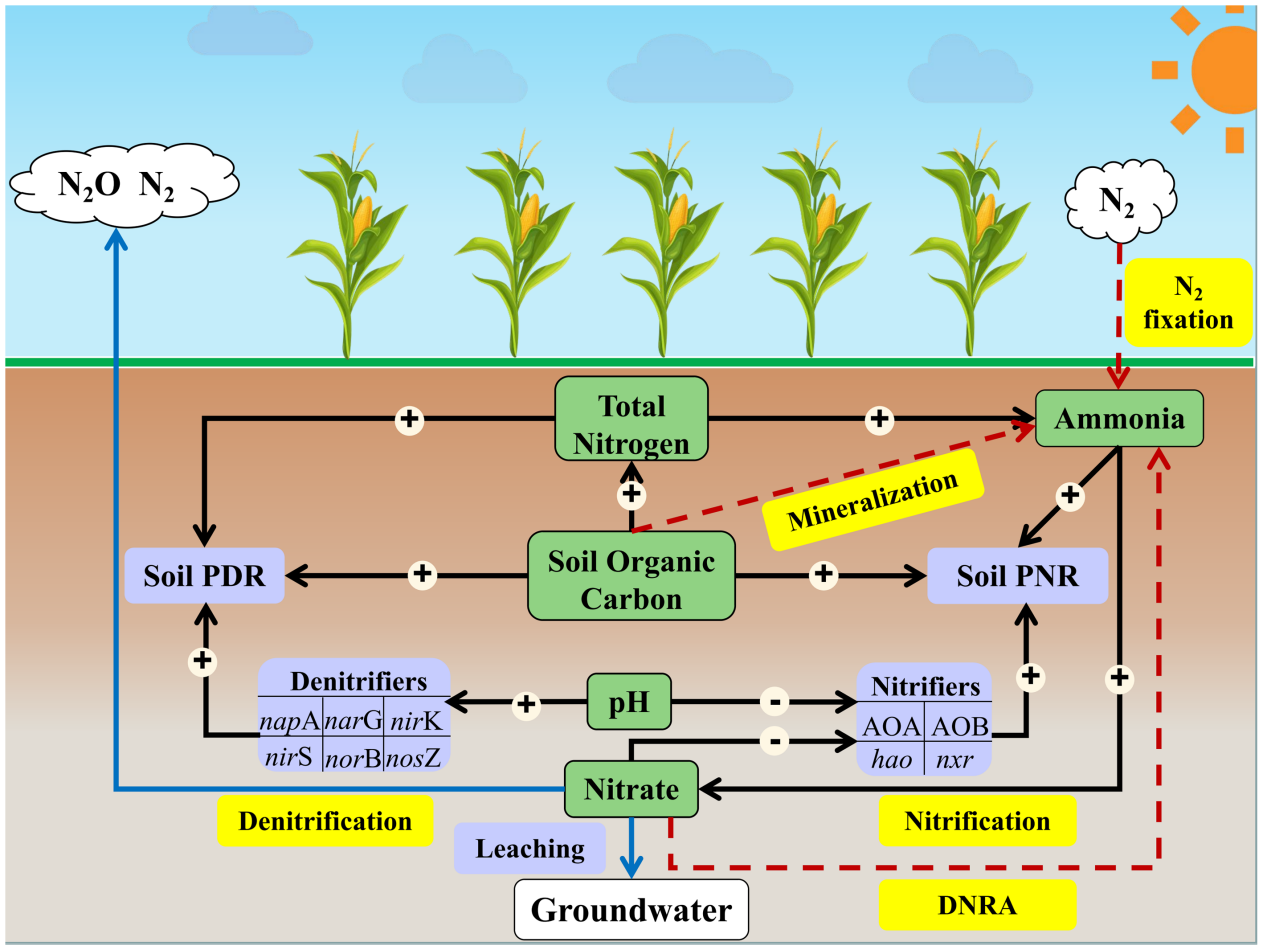
Conceptual diagram demonstrating how environmental and biological factors affect soil N transformation processes. The pathways of soil ammonia fixation are indicated by red arrows, and the pathways of nitrogen loss are indicated by blue arrows. The significant direct effects of N-cycling microorganisms and soil properties on soil potential nitrification rate and denitrification rate are indicated by black arrows. The symbols “+” and “−” in the circle associated with black arrows represent increasing and decreasing of effect, respectively, on the N transformation process. Soil PNR, soil potential nitrification rate. Soil PDR, soil potential nitrification rate.

The path model suggests that soil active nitrifiers (abundance of AOA and AOB *amo*A, *hao*, and *nxr*), SOC and NH_4_^+^ directly impacted soil PNR (*p <* 0.05; Figure 5), which accounted for 37.75% of the variation in soil PNR (Supplementary Figure S3A). Among all soil properties, soil TN showed the greatest indirect effect on soil PNR by exerting impacts on active nitrifiers and NH_4_^+^ (Supplementary Table S5). Soil active denitrifiers (abundance of *nap*A, *nar*G, *nir*K, *nir*S, *nor*B, and *nos*Z), TN and SOC were the dominant factors affecting soil PDR (*p <* 0.05; Figure 5), accounting for 42.23% of the variation in soil PDR (Supplementary Figure S3B). Soil pH showed greatest indirect effect (among all soil properties) on soil PDR by exerting impacts on active denitrifiers (Supplementary Table S5).

## Discussion

### Active N-cycling microorganisms in soils of long-term fertilization

Both of soil nitrification and denitrification would lead to the loss of soil nitrogen. Ammonia in soil is oxidized to nitrite and nitrate stepwise during nitrification, and nitrate can directly migrate with water leading to runoff of fertilizers (Ledoux et al., 2007; Wang et al., 2021). Meanwhile nitrate in soils can also be gradually reduced to nitrite, nitric oxide, nitrous oxide, and nitrogen gas during denitrification (Stevens et al., 1998), resulting to removal of nitrogen from soil ecosystems in gaseous forms. In this study, N chemical fertilizers (LCF and HCF treatments), particularly the HCF treatment (*p <* 0.05), reduced the active ammonia-oxidizer transcripts (both AOA and AOB; Figure 1). These results were in line with previous studies reporting negative relationships between inorganic N fertilizer and ammonia oxidizers in forest soils (Li and Gu, 2013; Wu et al., 2017a). The main reasons for the negative effects of N chemical fertilizers might include: (1) Although AOA and AOB are all autotrophic microbes, their cells are in close contact with heterotrophic microbial cells. NH_3_ produced by heterotrophic microorganisms could directly enter the cells of ammonia oxidizers, indicating that soil organic nitrogen mineralization may play an important role in influencing ammonia-oxidizers (De and Kowalchuk, 2001). Therefore, the decrease of SOC content in LCF and HCF treatments may lead to the decrease of ammonia-oxidizer transcripts (Table 1). (2) Fertilization decreased soil NH_4_^+^/NO_3_^−^ ratio (Table 1) and broke the balance between ammonia and nitrate, which inhibits the transcriptions of ammonia-oxidizers (Nadine et al., 2014; Du et al., 2021). Previous studies showed that the application of manure fertilization can appropriately enhance soil quality and provide abundant nutrients and suitable environment for microorganisms (Saggar et al., 2013; Lan et al., 2015; Mangalassery et al., 2018). In this study, compared to no fertilization, chemical fertilizer combined with manure fertilizer (CMF treatment) significantly decreased the transcription of *nir*K gene (regulating nitrite reduction). The transcript abundances of *nar*G gene (responsible for nitrate reduction) and *nos*Z gene (responsible for nitrous oxide reduction) were increased in CMF treatment (Figure 1). Based on the transcript abundances of the tested genes, *nap*A gene may be more important than *nar*G gene in nitrate reduction, while *nir*S gene may have contributed more to nitrite reduction than *nir*K gene in the soil samples (Figure 1). The relative less contribution of *nar*G and *nir*K genes to soil denitrification compared to *nap*A and *nir*S genes might be caused by the strong selective effect of soil pH (Priemé et al., 2002; Smith et al., 2007; Enrico et al., 2017; Zhang et al., 2017). Thus, the active *nos*Z gene might be the important functional group influencing soil denitrification in CMF treatment.

The chemical fertilizers (LCF and HCF) significantly decreased AOA *amo*A and *nos*Z transcripts at 0–20 cm soil horizon, and CMF treatment increased *nos*Z transcripts at 20–40 and 40–60 cm soil layers (*p < 0.05*; Supplementary Figure S1), which could be also attributed to low pH at top soil (Table 1). On the other hand, the negative effect of chemical fertilizer (LCF and/or HCF treatments) on AOA and AOB *amo*A transcripts and positive effect of CMF treatment on *nos*Z transcripts were more evident between different seasons rather than soil horizon (Supplementary Figure S2). And the PCA results showed that genes in both nitrification and denitrification were affected more significantly by season than soil horizon, which was inconsistent with Lipsewers et al. (2014) showing relatively stable AOA and AOB transcripts under seasonal cycle in marine coastal sediments. The difference may be due to the fertilization and cultivation activities in this study. Zhang et al. (2019) revealed that maize had a higher preference and absorbability for ammonia than nitrate, therefore soil nitrification was inhibited with maize growth in summer and autumn. The growth of maize and accumulation of plant litter in soil increased SOC and decreased NH_4_^+^/NO_3_^−^ ratios in summer and autumn (Table 1). The former can provide energy for denitrification, and the latter may promote nitrate reduction to balance soil ammonia and nitrate (Wei and Guodong, 2014; Liu et al., 2018), which may promote the growth of denitrifiers (Charles et al., 1999; Neumann et al., 2013).

### Potential N-cycling rates in soils of long-term fertilization

The mean values of soil potential nitrification and denitrification rates in the long-term fertilization soil were 2.68 and 33.27 mg kg^−1^ day^−1^, respectively (Figure 2). Soil PNR was highest in CK treatment and was significantly reduced in LCF treatment (*p <* 0.05; Supplementary Table S2). However, soil PDR was lowest in CK treatment and increased significantly in CMF treatment (*p <* 0.05; Supplementary Table S2). The lower PNR and higher PDR in fertilizer treatments were probably attributed to the SOC and TN contents, which were significantly correlated with PNR and PDR in this study (*p* < 0.01; Supplementary Table S4). The effect of fertilization on soil PNR in this study was inconsistent with previous studies conducted in other agricultural soils using similar method. For example, Jiang et al. (2013) detected that soil PNR ranged from 1.23 to 5.47 mg kg^−1^ h^−1^ and elevated more than 50% in organic fertilizer compared with no fertilizer in an acidic maize field after 10 years of fertilization. Chu et al. (2007) indicated that soil PNR under fertilization (mineral and/or organic fertilizer) were 13–21 times greater than that if no fertilizer treatment under a rotation of winter wheat and summer maize. The main reason for the different observations may be that the SOC content was increased after fertilization in these mentioned studies. By comparison, the SOC content was reduced (Table 1) by chemical fertilization treatments in our study, inhibiting soil nitrification under the lower C/N ratio (Cheng et al., 2017). Moreover, different from the decreased transcriptions of nitrifiers in chemical fertilization treatment (Figure 1), Jiang et al. (2013) and Chu et al. (2007) revealed that the abundance and diversity of nitrifiers were enhanced after fertilization, which may attribute to the higher temperature at these two sites (17.6 and 13.9°C) than in our study (7.9°C). Suitable temperature can increase the activity and abundance of nitrifiers (Rui et al., 2014). The effect of fertilization on soil PDR in our study was in line with the study by Pan et al. (2017), which indicates straw combined N chemical fertilizer could promote soil denitrification. Soil denitrification could be enhanced by manure combined N chemical fertilizer compared to sole N chemical fertilizer (Dambreville et al., 2006). Manure fertilization contains living microorganisms and enzymes, providing more comprehensive nutrients to microbes than chemical fertilization (Lily et al., 2018). The significant increase in SOC and TN contents in CMF treatment provided microbial community energy and substrate of denitrification (Wei and Guodong, 2014), therefore promoting the soil PDR.

In addition, both of the soil PNR and PDR were significantly higher in 0–20 cm soil horizon than those in 20–40 and 40–60 cm (*p <* 0.05), and the effect of fertilizer on soil PNR and PDR was also more pronounced in top soil (Figure 2). These results were not surprising, because soil properties, including TN, TC, NH_4_^+^, and NO_3_^−^ were highest in topsoil (0–20 cm) in this study (Table 1). Seasonal variation showed no significant effect on soil PNR, but soil PDR was significantly lower in spring than in summer and autumn (Supplementary Table S2). The results are inconsistent with Dai et al. (2020) reporting that elevated temperature enhanced nitrification and denitrification across global terrestrial ecosystems. However, the negative effect of low chemical fertilizer (LCF treatment) on soil PNR and the positive effect of chemical fertilizer combined with manure fertilizer (CMF treatment) on soil PDR were more evident in autumn than in spring and summer (Figure 2). These results may be explained by the following reasons, soil PNR was positively correlated with AOA *amo*A and *nxr* transcripts abundance (lowest in autumn), while soil PDR was positively correlated with *nap*A, *nir*S, and *nos*Z transcripts abundance (highest in autumn; Figure 1). Thus, we should pay more attention to soil denitrification process under long-term fertilization regimes, especially the application of manure fertilizers at top soil in autumn.

### Effects of environmental and biological factors on soil N-cycling

Soil PNR was significantly correlated to the abundances of AOA *amo*A and *nxr* transcripts (Supplementary Table S4), and SOC and TN were important factors influencing the active nitrifiers and soil nitrification (Figure 4). A study by Cai et al. (2021) showed that TN strongly affected the abundance and diversity of nitrifiers in different soil layers. However, the effect of TN on soil PNR in this study was mostly depended on its high indirect effect of explanatory variables, which was mediated through both NH_4_^+^ and the abundance of nitrifier transcripts (Supplementary Table S5; Figure 5; Supplementary Figure S3). The direct effects of NH_4_^+^ on soil PNR in long-term fertilization soil (Figure 5; *p <* 0.05) was consistent with the conclusions of previous studies in Tibetan wetlands and wheat soil under nitrogen fertilization conditions (Waghmode et al., 2019; Ma et al., 2020), suggesting that nitrogen availability was a vital factor of soil nitrification process. Ammonia as the substrate in nitrification directly influences nitrifiers and corresponding soil nitrification (Wu et al., 2017b). The direct impact of nitrifiers transcripts on soil PNR in this study (Figure 5; Supplementary Table S5) also indicated that soil properties affect N-cycling by influencing soil microorganisms (Tatariw et al., 2013). Thus, the variation of SOC, TN, and NH_4_^+^ contents in soils affected the transcription of key nitrifiers genes (AOA *amo*A and *nxr*) and eventually regulated the soil PNR.

Similar to nitrification, the abundances of denitrifiers (*nap*A, *nir*S, and *nos*Z) significantly correlated to soil PDR (Supplementary Table S4), and TN and SOC were also the primary factors of influencing soil PDR. Both denitrifiers and soil properties (TN and SOC) showed the direct effects in soil denitrification, with the highest effect in TN content (Figure 5; *p <* 0.05; Supplementary Table S5). Previous studies showed that soil denitrification was affected by many environmental factors, especially the SOC content (Xiong et al., 2017; Ma et al., 2020), as the decomposition of organic matter could contribute electrons and energy to the denitrification process (Ahn, 2006). The direct impact of abundance of denitrifiers transcripts on soil PDR (Figure 5; Supplementary Table S5) was inconsistent with previous studies which showed no relationship between denitrifiers abundances and denitrification rate (Cindy et al., 2015; Liu et al., 2018). One possible explanation for the contradictory results is that the abundance of denitrifiers used in this study was based on RNA level and had better representation of the activity of denitrification than DNA level (Hommes et al., 2001). Soil pH is considered one of the limiting factors for the soil denitrification (Liu et al., 2010), and it was reported that changes in soil pH could alter the ionized state of nutrients, reducing their effectiveness at utilization by microorganisms (Joanne et al., 2014). In this study, soil pH showed the highest indirect effect on soil PDR, by influencing the abundance of denitrifier transcripts (Supplementary Figure S3). The influence of soil pH on denitrification could be attributed to the significant correlation between soil pH and key denitrifiers (*nap*A, *nir*S, and *nos*Z; Supplementary Table S4). Similar results were also reported in previous studies in coastal wetlands and grassland soils (Liu et al., 2010; Cindy et al., 2015; Zhe et al., 2015).

Collectively, fertilization can significantly influence the functional gene transcription of soil N-cycling microorganisms across different seasons and soil horizons thereby influencing nitrogen cycle in soil. However, the soil samples of our study were collected from only one field, and it is necessary to investigate whether the conclusions of this work are valid at larger scales. Another limitation of our study is that we only tested 10 functional genes associated with N cycling, and more N-cycling genes need to be investigated for better understanding microbial mediated N cycling processes under fertilization practices. For example, comammox, anaerobic ammonia oxidation (Anammox) bacteria, *nos*Z II, c*nor*B, and n*or*Z could be included in future studies. In this study, the abundances of N-cycling functional genes were examined only at the cDNA level, and more molecular biological methods, such as DNA-SIP, metabolome, and proteomics, should be applied to reflect the dynamics of active microorganisms and soil ecosystem functioning. For example, the association between functional microorganisms and nitrogen transformation process could be better established through the integration of amplicon sequencing and qPCR data.

## Conclusion

Long-term application of chemical fertilizers decreased soil PNR, and chemical fertilizer combined with manure fertilizer (CMF) increased soil PDR. Soil organic carbon have direct on both soil PNR and PDR, and total nitrogen and pH can regulate active nitrifiers and denitrifiers and affect the corresponding PNR and PDR in soil indirectly. Fertilization also significantly affected soil properties and transcriptions of N-cycling microorganisms, and these impacts may vary across different seasons and soil horizons. The active microbes containing AOA *amo*A and *nxr* genes have key roles in soil nitrification, while *nap*A, *nir*S, and *nos*Z genes were important in regulating soil denitrification.

## Data availability statement

The raw data supporting the conclusions of this article will be made available by the authors, without undue reservation.

## Author contributions

FW, XL, and LL wrote the manuscript. FW and FD analyzed the data. FW, LR, and ML carried out the research. TA and SL collected the soil samples. LL and JW designed the project. All authors contributed to the article and approved the submitted version.

## Funding

This work was supported by the National Key Research and Development Program of China (Grant number: 2021YFD1500205), Natural Science Foundation of Liaoning Province (Grant number: 2019-MS-271) and the Science Project of Liaoning Province (Grant number: 2020JH2/10200034).

## Conflict of interest

The authors declare that the research was conducted in the absence of any commercial or financial relationships that could be construed as a potential conflict of interest.

## Publisher’s note

All claims expressed in this article are solely those of the authors and do not necessarily represent those of their affiliated organizations, or those of the publisher, the editors and the reviewers. Any product that may be evaluated in this article, or claim that may be made by its manufacturer, is not guaranteed or endorsed by the publisher.

## Supplementary material

The Supplementary material for this article can be found online at: https://www.frontiersin.org/articles/10.3389/fmicb.2022.1021080/full#supplementary-material
