# Supplementary material for: The active functional microbes contribute differently to soil nitrification and denitrification potential under long-term fertilizer regimes in North-East China
Source: Front Microbiol. 2022 Oct 3;13:1021080. doi: 10.3389/fmicb.2022.1021080 (PMC9576102; doi:10.3389/fmicb.2022.1021080)
Supplement: Supplementary file 1 [file Data_Sheet_1.docx]

**Supplementary material:**

**Table S1 The primers used for quantitative real-time PCR.**

| **Primer set** | **Target gene** | **Amplicon length** | **Encoding protein** | **References** |
| --- | --- | --- | --- | --- |
| CamoA-19F (ATGGTCTGGYTWAGACG) | AOA-  *amo*A | 629 bp | Ammonia monooxygenase | (Könneke et al, 2005) |
| CamoA-616R (GCCATCCABCKRTANGTCCA) |  |  |  |  |
| amoA-1F (GGGGTTTCTACTGGTGGT) | AOB-  *amo*A | 491 bp | Ammonia monooxygenase | (Rasche et al, 2011) |
| amoA-2R (CCCCTCKGSAAAGCCTTCTTC) |  |  |  |  |
| hao F1 (TGCGTGGARTGYCAC) | *hao* | 992 bp | Hydroxylamine oxidation | (Schmid et al, 2008) |
| hao R3 (AGRTARGAKYSGGCAAA) |  |  |  |  |
| nxrA-F (CAGACCGACGTGTGCGAAG) | *nxr* | 322 bp | Nitrite oxidation | (Poly et al, 2008) |
| nxrA-R (TCYACAAGGAACGGAAGGTC) |  |  |  |  |
| narG-1960m2f (TA(CT)GT(GC)GGGCAGGA(AG)AAACTG) | *nar*G | 100 bp | Nitrate reductase alpha | (López et al, 2004) |
| narG-2050m2r (CGTAGAAGAAGCTGGTGCTGTT) |  |  |  |  |
| napA1F (TCTGGACCATGGGCTTCAACC) | *nap*A | 870 bp | Periplasmi nitrate reductase | (Bru et al, 2007) |
| napA2F (ACGACGACCGGCCAGCGCAG) |  |  |  |  |
| nirK583F (TCATGGTGCTGCCGCGKGACGG ) | *nir*K | 326 bp | Nitrite reductase（Cu） | (Yan et al, 2003) |
| nirK909R (GAACTTGCCGGTKGCCCAGAC) |  |  |  |  |
| nirScd3Af (GT(C,G)AACGT(C,G)AAGGA(A,G)AC(C,G)GG) | *nir*S | 425 bp | Nitrite reductase（cdl） | (Throbäck et al, 2004) |
| nirSR3cd (GA(C,G)TTCGG(A,G)TG(C,G)GTCTTGA) |  |  |  |  |
| norB2F (GGNCAYCARGGNTAYGA ) | *nor*B | 262 bp | NO reductase | (Braker and Tiedje, 2003) |
| norB5R (ACCCANAGRTGNACNACCCACCA) |  |  |  |  |
| nosZ 1527F (CGCTGTTCHTCGACAGYCA ) | *nos*Z | 250 bp | N_2_O reductase | (Rich et al, 2003) |
| nosZ 1773R (ATRTCGATCARCTGBTCGTT) |  |  |  |  |

**Table S2 The potential N-cycling rates averaged fertilization, soil horizons and seasons.**

|  | **PNR**  **(mg kg^-1^ d^-1^)** | **PDR**  **(mg kg^-1^ d^-1^)** |
| --- | --- | --- |
| **Fertilizer (F)** | | |
| CK | 3.24 a | 33.06 b |
| LCF | 1.93 b | 33.30 ab |
| HCF | 2.76 a | 33.17 b |
| CMF | 2.77 a | 33.55 a |
| **Soil horizons (H)** | | |
| 0-20 cm | 3.56 a | 33.97 a |
| 20-40 cm | 2.26 b | 33.01 b |
| 40-60 cm | 2.21 b | 32.84 b |
| **Seasons (S)** | | |
| Spring | 2.61 | 32.97 b |
| Summer | 2.89 | 33.29 a |
| Autumn | 2.53 | 33.55 a |

^a^ The different letters indicate significant differences based on LSD multiple comparisons test *(P<0.05*).

**Table S3 Permutational multivariate analysis of variance (PERMANOVA).**

| **Factors** | **Nitrification** | **AOA**  ***amo*A** | **AOB**  ***amo*A** | ***hao*** | ***nxr*** | **Denitrification** | ***nap*A** | ***nar*G** | ***nir*K** | ***nir*S** | ***nor*B** | ***nos*Z** |
| --- | --- | --- | --- | --- | --- | --- | --- | --- | --- | --- | --- | --- |
| **Fertilizer (F)** | | | | | | | | | | | | |
| R^2^ | 0.0450 | 0.1114 | 0.0474 | 0.0378 | 0.0292 | 0.0254 | 0.0245 | 0.0428 | 0.0796 | 0.0056 | 0.0103 | 0.0540 |
| P | 0.001 | 0.001 | 0.003 | 0.018 | 0.005 | 0.002 | 0.001 | 0.004 | 0.013 | 0.038 | 0.507 | 0.001 |
| **Soil horizons (H)** | | | | | | | | | | | | |
| R^2^ | 0.1195 | 0.1368 | 0.0018 | 0.0185 | 0.1566 | 0.0094 | 0.0011 | 0.0329 | 0.0240 | 0.0028 | 0.0091 | 0.0159 |
| P | 0.001 | 0.001 | 0.787 | 0.098 | 0.001 | 0.120 | 0.118 | 0.012 | 0.166 | 0.115 | 0.345 | 0.047 |
| **Seasons (S)** | | | | | | | | | | | | |
| R^2^ | 0.2973 | 0.3851 | 0.4664 | 0.1203 | 0.0073 | 0.3446 | 0.8807 | 0.0892 | 0.0287 | 0.8068 | 0.2526 | 0.1763 |
| P | 0.001 | 0.001 | 0.001 | 0.001 | 0.162 | 0.001 | 0.001 | 0.001 | 0.117 | 0.001 | 0.001 | 0.001 |
| **F*H** | | | | | | | | | | | | |
| R^2^ | 0.0712 | 0.0301 | 0.0744 | 0.1138 | 0.1382 | 0.1386 | 0.0213 | 0.2478 | 0.0792 | 0.0492 | 0.1386 | 0.1743 |
| P | 0.001 | 0.001 | 0.001 | 0.001 | 0.001 | 0.001 | 0.001 | 0.001 | 0.056 | 0.001 | 0.001 | 0.001 |
| **F*S** | | | | | | | | | | | | |
| R^2^ | 0.1359 | 0.1534 | 0.0942 | 0.1533 | 0.0175 | 0.0893 | 0.0201 | 0.1309 | 0.0945 | 0.0260 | 0.0724 | 0.1102 |
| P | 0.001 | 0.001 | 0.002 | 0.001 | 0.197 | 0.001 | 0.001 | 0.001 | 0.026 | 0.001 | 0.016 | 0.001 |
| **H*S** | | | | | | | | | | | | |
| R^2^ | 0.0559 | 0.0632 | 0.0217 | 0.0838 | 0.0732 | 0.0295 | 0.0041 | 0.0401 | 0.0395 | 0.0057 | 0.0379 | 0.0379 |
| P | 0.001 | 0.001 | 0.193 | 0.002 | 0.001 | 0.009 | 0.003 | 0.026 | 0.198 | 0.061 | 0.072 | 0.009 |
| **F* H*S** | | | | | | | | | | | | |
| R^2^ | 0.1476 | 0.0587 | 0.0469 | 0.1760 | 0.4355 | 0.1684 | 0.0288 | 0.1678 | 0.1847 | 0.0588 | 0.1748 | 0.2460 |
| P | 0.001 | 0.001 | 0.331 | 0.002 | 0.001 | 0.001 | 0.001 | 0.001 | 0.006 | 0.001 | 0.001 | 0.001 |

**Table S4 Pearson’s correlation coefficient between the microbial abundances and rates of N cycling and environmental factors.**

| Genes | TN | SOC | NH_4_^+^ | NO_3_^-^ | pH | PNR | PDR |
| --- | --- | --- | --- | --- | --- | --- | --- |
| AOA-*amo*A | 0.354** | 0.429** | 0.221* | 0.157 | -0.108 | 0.405** | - |
| AOB-*amo*A | 0.066 | 0.026 | -0.084 | -.570** | 0.301** | -0.032 | - |
| *hao* | -0.098 | -0.074 | 0.097 | 0.126 | -0.174 | 0.093 | - |
| *nxr* | 0.219* | 0.264** | 0.229* | 0.178 | -0.175 | 0.174* | - |
| *nap*A | 0.113 | 0.100 | -0.324** | -0.331** | 0.432** | - | 0.292** |
| *nar*G | -0.078 | -0.105 | -0.065 | 0.046 | 0.123 | - | -0.210* |
| *nir*K | -0.152 | -0.189* | 0.007 | -0.042 | 0.044 | - | -0.122 |
| *nir*S | 0.097 | 0.073 | -0.275** | -0.280** | 0.354** | - | 0.241* |
| *nor*B | -0.076 | 0.022 | -0.067 | 0.097 | -0.057 | - | -0.150 |
| *nos*Z | 0.101 | 0.108 | -0.182 | 0.058 | 0.331** | - | 0.234* |
| PNR | 0.550** | 0.554** | 0.048 | -0.025 | 0.062 | - | - |
| PDR | 0.720** | 0.754** | 0.303** | 0.229* | -0.176 | - | - |

^a^ * and ** indicate significance at P=0.05 and 0.01, respectively.

^b^ - indicate no detect.**Table S5 Total, direct, and indirect effects of explanatory variables on soil potential N-cycling rates.**

|  | Potential Nitrification rate | | | Potential Denitrification rate | | |
| --- | --- | --- | --- | --- | --- | --- |
|  | **Direct** | **Indirect** | **Total** | **Direct** | **Indirect** | **Total** |
| TN | 0.0764 | 0.4445 | 0.5209 | 0.6470 | 0.0142 | 0.6612 |
| SOC | 0.2942 | 0.2091 | 0.5033 | 0.2989 | 0.0819 | 0.3808 |
| NH_4_^+^ | 0.5458 | -0.0888 | 0.4570 | 0.0241 | 0.0549 | 0.0790 |
| NO_3_^-^ | 0.066 | 0.1331 | 0.1991 | 0.0482 | 0.0460 | 0.0942 |
| pH | 0.0040 | -0.0610 | -0.0570 | 0.0382 | -0.1170 | -0.0788 |
| Nitrifiers | 0.2096 | - | 0.2096 | - | - | - |
| Denitrifiers | - | - | - | 0.2732 | - | 0.2732 |

**Fig. S1** The impacts of fertilization on the abundance of active N-cycling microorganisms in different soil horizons. The significant differences between samples were represented with different letters as shown besides each bar (at P ≤ 0.05 according to the LSD multiple comparisons test).

**Fig. S2** The impacts of fertilization on the abundance of active N-cycling microorganisms in different seasons. The significant differences between samples were represented with different letters as shown besides each bar (at *P* ≤ 0.05 according to the LSD multiple comparisons test).

**
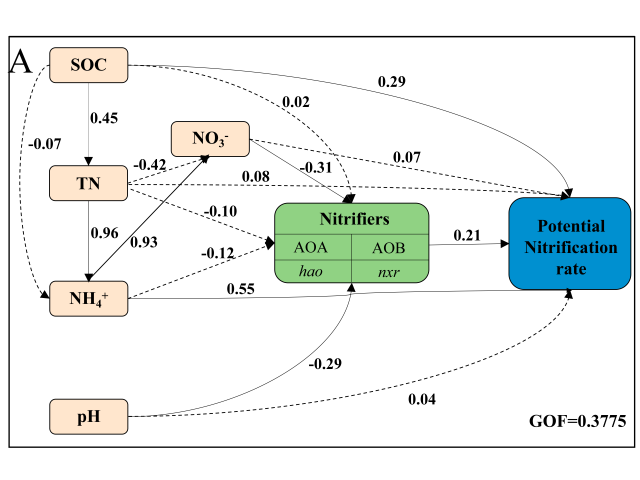

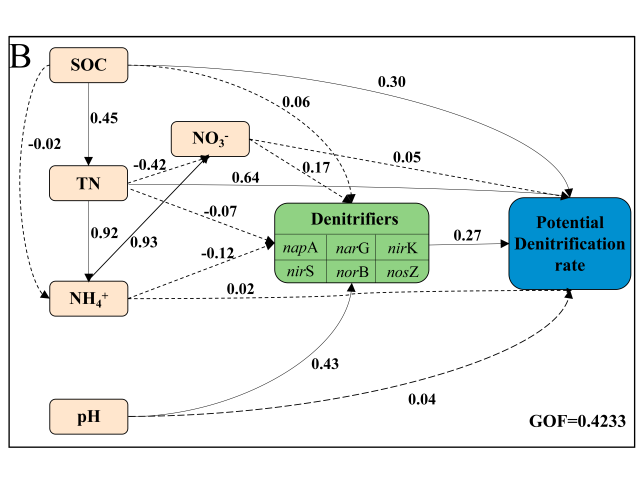
**

**Fig. S3** Effects of N-cycling microorganisms and soil properties on soil potential nitrification rate (A) and denitrification rate (B). The solid and dashed lines represent significant (*p < 0.05*) and insignificant effects, respectively. The numbers nearby the lines are path coefficients. The direction of arrows indicates causal relationships.

**References**

Braker, G., Tiedje, J.M., 2003. Nitric oxide reductase (nor B) genes from pure cultures and environmental samples. Appl. Environ. Microbiol. 69: 3476-3483. https://doi.org/10.1128/AEM.69.6.3476-3483.2003.

Bru, D., Sarr, A., Philippot, L., 2007. Relative Abundances of Proteobacterial Membrane-Bound and Periplasmic Nitrate Reductases in Selected Environments. Appl. Environ. Microb. 73, 5971-5974. https://doi.org/10.1128/AEM.00643-07.

Könneke, M., Bernhard, A.E., Walker, C.B., Waterbury, J.B., Stahl, D.A., 2005. Isolation of an autotrophic ammonia-oxidizing marine archaeon. Nature. 437, 543-546. https://doi.org/10.1038/nature03911.

López-Gutiérrez, J.C., Sonia, H., Stéphanie, H., Martin-Laurent, F., Catroux, G., 2004. Quantification of a novel group of nitrate-reducing bacteria in the environment by real-time PCR. J. Microbiol. Meth. 57, 399-407. https://doi.org/10.1016/j.mimet.2004.02.009.

Poly, F., Wertz, S., Brothier, E., Degrange, V., 2008. First exploration of Nitrobacter diversity in soils by a PCR cloning-sequencing approach targeting functional gene nxr A. Fems Microbiol. Lett. 63, 132-140. https://doi.org/10.1111/j.1574-6941.2007.00404.x.

Rasche, F., Knapp, D., Kaiser, C., Koranda, M., Kitzler, B., Zechmeister-Boltenstern, S., Richter, A., Sessitsch, A., 2011. Seasonality and resource availability control bacterial and archaeal communities in soils of a temperate beech forest. ISME J. 5, 89-402. https://doi.org/10.1038/ismej.2010.138.

Rich, J.J., Heichen, R.S., Bottomley, P.J., Cromack, K., Myrold, D.D. 2003. Community composition and functioning of denitrifying bacteria from adjacent meadow and forest soils. Appl. Environ. Microbiol. 69, 5974-5982. https://doi.org/10.1128/AEM.69.10.5974-5982.2003.

Schmid, M.C., Hooper, A.B., Klotz, M.G., Woebken, D., Lam, P., Kuypers, M.M.M, Pommerening-Roeser, A., Camp, H., Jetten, M.S.M., 2008. Environmental detection of octahaem cytoc- hrome chydroxylamine/hydrazine oxidoreductase genes of aerobic and anaerobic ammonium-oxidiz- ing bacteria. Environ. Microbiol. 10, 3140-3149. https://doi.org/10.1111/j.1462-2920.2008.01732.x.

Throbäck, I.N., Enwall, K., Jarvis, Å., Hallin, S., 2004. Reassessing PCR primers targeting nirS, nirK and nosZ genes for community surveys of denitrifying bacteria with DGGE. Fems Microbiol. Lett. 49, 401-417. https://doi.org/10.1016/j.femsec.2004.04.011.

Yan, T.F., Fields, M.W., Wu, L.Y., Zu, Y.G., Tiedje, J.M., Zhou, J.Z., 2003. Molecular diversity and characterization of nitrite reductase gene fragments (nirK and nirS) from nitrate- and uranium-contaminated groundwater. Environ. Microbiol. 5, 13-24. https://doi.org/10.1046/j.1462-2920.2003.00393.x.
